# Triterpenoids from the Herbs of *Salicornia bigelovii*

**DOI:** 10.3390/molecules201119695

**Published:** 2015-11-12

**Authors:** Yu Shan, Huan Li, Fuqin Guan, Yu Chen, Min Yin, Ming Wang, Xu Feng, Qizhi Wang

**Affiliations:** 1 Jiangsu Key Laboratory for Bioresources of Saline Soils, Institute of Botany, Jiangsu Province and Chinese Academy of Sciences, Nanjing 210014, China; attilayu@hotmail.com (Y.S.); lihuan405@sina.cn (H.L.); tube1031aaa@126.com (F.G.); chenyu.1007@163.com (Y.C.); epmin@sohu.com (M.Y.); wangmingwm0208@sina.com (M.W.); fengxucnbg@cnbg.net (X.F.); 2 Jiangsu Provincial Platform for Conservation and Utilization of Agricultural Germplasm, Nanjing 210014, China

**Keywords:** *Salicornia bigelovii* Torr., nortriterpenoid saponins, antifungal activity

## Abstract

A new nortriterpene saponin, 3-*O*-β-d-glucuronopyranosyl-30-norolean-12,20(29)-dien-23-oxo-28-oic acid, namely bigelovii D (**11**), was isolated from the hydroalcoholic extract of herbs of *Salicornia bigelovii* along with 10 known saponins (**1**–**10**). Their chemical structures were identified on the basis of spectroscopic analyses including two-dimensional NMR and a comparison with literature data. Some of these compounds showed potent antifungal activities *in vitro*. Compounds **3**, **4**, **5**, **6**, **7**, **10** and **11** demonstrated potent inhibitory activities against *Colletotrichum gloeosporioides* and compound **11** displayed broad-spectrum inhibitory activity against *Alternaria alternata*, *A. solani*, *Botrytis cinerea*, *C. gloeosporioides*, *Fusarium graminearum*, *F. verticilloides*, *Thanatephorus cucumeris* and *Sclerotinia sclerotiorum*, with EC_50_ values ranging from 13.6 to 36.3 μg/mL.

## 1. Introduction

*Salicornia bigelovii* is a salt-tolerant land plant that grows on salt marshes and muddy seashores and is widespread in tropical and subtropical North America, Asia and Europe. It belongs to *Salicornia* (Chenopodiaceae), which is a genus of annual, apparently leafless, halophytic herbs that have articulated and succulent stems [1]. It was reviewed as an oil seed crop and seasoned vegetable with direct seawater irrigation [2]. Recently, the consumption of this plant as functional foods and medicinal plants has increased considerably because of their beneficial effects in the treatment of constipation, obesity, diabetes and cancer [3]. During our ongoing screening for biologically active constituents [1], there were no previous reports of the inhibitory activity against plant pathogens of extracts from *S. bigelovii*. It has been reported that *S. bigelovii* contains a diverse range of bioactive phytochemicals, including chlorogenic acid derivatives, quercetin glucosides and triterpenoid saponins [1,4,5,6]. In order to examine its chemical constituents further, the present study describes the isolation of 11 nortriterpene and triterpene analogues, including a new nortriterpene saponin, 3-*O*-β-d-glucuronopyranosyl-30-norolean-12,20(29)dien-23-oxo-28-oic acid, namely bigelovii D, as well as their inhibitory activities against plant pathogens.

## 2. Results and Discussion

**Figure 1:**
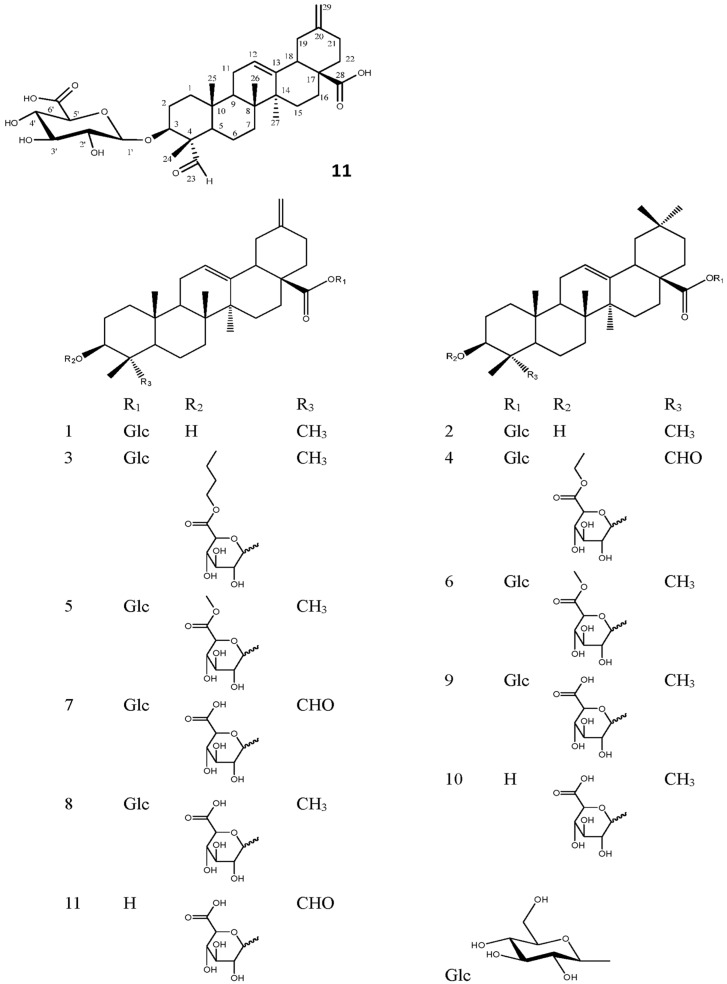
Structures of compounds **1**–**11**.

The powder of the herbs was extracted using 80% ethanol, and the concentrated extracts were partitioned successively with petroleum ether (PE), ethyl acetate (EtOAc) and *n*-BuOH. Column chromatography of the *n*-BuOH-soluble fraction yielded one new compound (**11**) and 10 known compounds (**1**–**10**) (Figure 1).

**Figure 2:**
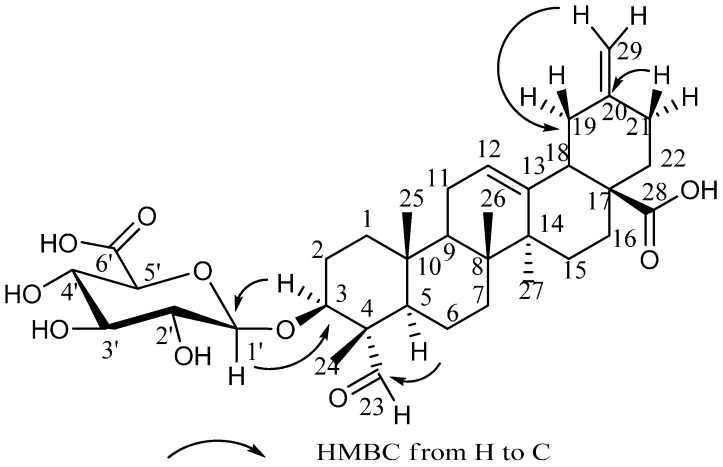
Key HMBC correlations for compounds **11**.

Compound (**11**) was obtained as a white amorphous powder with a positive optical rotation (${\left[\alpha \right]}_{\text{D}}^{20}$ +27.9 (*c* 0.479, MeOH)). The IR spectrum of **11** showed absorption bands at 3412, 1705 and 1645 cm^−1^ ascribable to hydroxy, carboxy and olefin functions. The negative-ion high-resolution electrospray ionization mass spectrum (HR-ESI-MS) analysis revealed the molecular formula of **11** to be C_35_H_50_O_10_, showing a deprotonated molecule [M − H]^−^ at *m*/*z* 629.3261 (calc. for C_35_H_49_O_10_: 629.3326). The ^1^H-NMR and ^13^C-NMR (Table 1) spectra of **11**, which were assigned by various NMR experiments showed signals assignable to four methyls [δ_H_ 0.77, 0.88, 1.26, 1.28 (3H each, all s, H_3_-25, 26, 27, 24)], one methine bearing an oxygen function [δ_H_ 4.17 (1H, dd, *J* = 4.5, 11.6 Hz, H-3)], three olefinic protons [δ_H_ 4.77, 4.73 (1H each, both s like, H_2_-29), 5.43 (1H, br d like, H-12)], an aldehyde group [δ_H_ 9.73 (1H, s)]. These data were correlated with information from ^13^C-NMR spectrum. Two signals at δ_H_ 122.7 and 144.2 ascribable to C-12 and C-13 suggested a ∆^12^ oleanene skeleton. Four methyl resonances (δ_C_ 10.4, 15.5, 17.2, 26.1), four sp2-hybridized carbon signals (δ_C_ 122.7, 144.2, 149.0, 107.0) and an aldehyde group (δ_C_ 206.8), and two carboxy carbon (δ_C_ 179.3 and 179.3, C-28 and C-6′) were shown in its ^13^C-NMR spectrum. The ^13^C-NMR spectrum of **11** displayed 35 carbon resonances of which 29 carbon signals were assigned to a noroleanic-acid-type triterpene moiety and six to a monosaccharide portion. The proton and carbon signals of **11** in the ^1^H- and ^13^C-NMR spectra resembled those of glucopyranosiduronic acid (6′-COOH). This assumption was supported by a set of 2D NMR experiments that provided decisive information concerning the structure of compound **11**. The chemical shifts of all the individual protons and carbons of the sugar unit were assigned on the basis of ^1^H-^1^H COSY spectra analysis. The β-glucopyranosyluronic group, which was deduced from its ^3^*J*_H-1, H-2_ coupling constant of 7.8 Hz, was placed at C-3 of the aglycon on the basis of the HMBC correlation between the anomeric proton at δ_H_ 4.84 (1H, d, *J* = 7.8 Hz) and the C-3 carbon resonance at δ_C_ 82.0. In the heteronuclear multiple bond correlations spectroscopy (HMBC) experiment (Figure 2), long-range correlations were observed between the following protons and carbons: H-5 and C-4, 10; H-12 and C-9, 14; H-18 and C-12, 13; H-23 and C-4, 24; H-24 and C-3, 4, 5, 23; H-25 and C-9; H-26 and C-7, 9; H-27 and C-9, 13, 15; H-29 and C-19, 21. The configuration of the glucuronic unit was established as D after hydrolysis of 1 N HCl, trimethylsilation, and determination of retention time by GC. On the basis of this evidence, the structure of **11** was characterized as shown. Thus the structure of **11** was deduced as 3-*O*-β-d-glucuronopyranosyl-30-norolean-12,20(29)-dien-23-oxo-28-oic acid and named bigelovii D.

The 10 known compounds were identified as pfaffine B (**1**), oleanolic acid 28-*O*-β-d-glucopyranoside (**2**), bigelovii A (**3**), 3-*O*-[(6’-ethyl-ester)-d-glucuronopyranosyl]-oleanolicacid-28-*O*-β-d-glucopyranoside (**4**), 3-*O*-[(6’-methyl-ester)-β-d-glucuronopyranosyl]-30-norolean-12,20(29)-dien-28-oic-28-*O*-β-d-glucuronopyranosyl ester (**5**), chikusetsusaponin IVa methyl ester (**6**), bigelovii B (**7**), boussingoside A2 (**8**), 3-*O*-β-d-glucuronopyranosyl-oleanolic acid 28-*O*-β-d-glucopyranoside (**9**) and 3-*O*-β-d-glucuronopyranosyl-oleanolic acid (**10**), respectively, by comparing their physical data (m.p., NMR, MS, and IR) with those reported in the literature [1,7,8,9,10,11].

**Table 1:** NMR spectroscopic data for compounds **11** in pyridine-*d*_5_ (^1^H: 500 MHz, ^13^C: 125 MHz).

No.	^1^H-NMR [δ (ppm), mult., *J* (Hz)]	^13^C-NMR [δ (ppm)]	DEPT	^1^H-^1^HCOSY	HMBC
1α	0.88 (1H, m)	38.1 (t)	CH_2_	H-2	
1β	1.39 (1H, m)				
2α	1.85 (1H, m)	25.1 (t)	CH_2_	H-3, H-1	
2β	2.23 (1H, m)				
3	4.17 (1H, dd, *J* = 4.5, 11.6)	82.0 (d)	CH	H-2	C-1, C-2
4		55.4 (s)	C		
5	1.31 (1H, m)	47.7 (d)	CH	H-23	C-4, C-10
6α	1.35 (1H, m)	20.4 (t)	CH_2_	H-7	
6β	1.01 (1H, m)				
7α	1.39 (1H, m)	32.4 (t)	CH_2_	H-6	
7β	1.11 (1H, m)				
8		40.0 (s)	C		
9	1.63 (1H, t, *J* = 7.7, 8.7)	47.9 (d)	CH		
10		36.1 (s)	C		
11α	1.86 (1H, m)	23.6 (t)	CH_2_	H-12	
11β	1.86 (1H, m)				
12	5.44 (1H, br s)	122.7 (d)	CH	H-11	C-9, C-14
13		144.2 (s)	C		
14		42.1 (s)	C		
15α	1.13 (1H, m)	28.2 (t)	CH_2_	H-16	
15β	2.10 (1H, m)				
16α	2.16 (1H, m)	23.7 (t)	CH_2_	H-15	C-18
16β	2.03 (1H, m)				
17		47.0 (s)	C		
18	3.18 (1H, dd, *J* = 4.4)	47.8 (d)	CH	H-19	C-12, C-13
19α	2.62 (1H, t, *J* = 13.7, 13.3)	41.9 (t)	CH_2_	H-18	C-18, C-20, C-29
19β	2.22 (1H, m)				
20		149.0 (s)	C		
21α	1.16 (1H, m)	30.4 (t)	CH_2_	H-22	C-20
21β	2.28 (1H, m)				
22α	1.87 (1H, m)	38.3 (t)	CH_2_	H-21	C-20
22β	2.18 (1H, m)				
23	9.73 (1H, s)	206.8 (s)	CHO		C-4, C-24
24	1.27 (3H, s)	10.4 (q)	CH_3_		C-3, C-4, C-5, C-23
25	0.77 (3H, s)	15.5 (q)	CH_3_		C-9
26	0.89 (3H, s)	17.2 (q)	CH_3_		C-7, C-9
27	1.24 (3H, s)	26.1 (q)	CH_3_		C-9, C-13, C-15
28		179.3 (s)	C		
29α	4.78 (1H, s)	107.0 (t)	CH_2_		C-19, C-21
29β	4.73 (1H, s)				
1’	4.84 (1H, d, *J* = 7.8)	105.0 (d)	CH	H-2’	C-3
2’	3.88 (1H, m)	75.0 (d)	CH	H-1’	C-1’
3’	4.17 (1H, m)	78.0 (d)	CH	H-4’,	C-4’, C-5’
4’	4.21 (1H, m)	73.3 (d)	CH	H-3’, H-5’	C-3’
5’	4.27 (1H, m)	77.8 (d)	CH	H-4’	C-4’
6’		179.3 (s)	C		

The **6** nortriterpenoid saponins and **5** triterpenoid saponins were evaluated for their inhibitory activities on plant pathogenic fungus. As showed in Table 2, compounds **3**, **4**, **5**, **6**, **7**, **10** and **11** demonstrated potent inhibitory activities against *Colletotrichum gloeosporioides*, while compounds **1**, **2**, **8** and **9** were inactive. The compound **11** was the most active of these compounds, with 69.21% inhibition at 100 μg/mL after 72 h. To determine the broad-spectrum inhibitory activity of compound **11** against plant pathogenic fungi, it was tested against *Alternaria alternata*, *A. solani*, *Botrytis cinerea*, *C. alterosporioides*, *Fusarium graminearum*, *F. verticilloides*, *Thanatephorus cucumeris*, *Sclerotinia sclerotiorum*. As shown in Table 3, compound **11** exhibited inhibitory activity against all of the tested pathogenic fungi and showed the highest activity against *B. cinerea* and *T. cucumeris*, with EC_50_ values of 13.6 μg/mL and 13.9 μg/mL respectively. The EC_50_ values of compound **11** against the other six pathogenic fungi were between 19.4 and 36.3 μg/mL. To the best of our knowledge, this study represented the first report on significant antifungal potential of saponins of *S. bigelovii*.

**Table 2:** Inhibitory activities of compounds **1**–**11** (100 mg/mL) against *C. gloeosporioides.*

Pathogenic Fungi	Inhibition after 72 h (%)											
	1	2	3	4	5	6	7	8	9	10	11	AMB
*C. gloeosporioides*	−2.53	−5.24	27.61	24.15	13.65	14.77	33.27	−1.90	−1.48	7.54	69.21	73.89

**Table 3:** Inhibitory activities of compound **11** against seven pathogenic fungi (EC_50_, μg/mL).

Pathogenic Fungi	11
*A. alternata*	31.8 ± 1.3
*A. solani*	23.3 ± 1.5
*B. cinerea*	13.6 ± 0.6
*C. gloeosporioides*	34.0 ± 0.9
*F. graminearum*	28.8 ± 1.7
*F. verticilloides*	19.4 ± 1.1
*T. cucumeris*	13.9 ± 0.7
*S. sclerotiorum*	36.3 ± 2.1

## 3. Materials and Methods

### 3.1. General Experimental Procedures

Melting points were measured using a XT-4 Boetius micro melting point apparatus (Beijing, China). Specific rotations were obtained on a Perkin-Elmer 341 digital polarimeter (Waltham, MA, USA). IR spectra were taken as the KBr discs on a Thermo Nicolet Nexus 870 FT-IR E.S.P. spectrometer (Beijing, China). High resolution electrospray ionization mass spectroscopy (HR-ESI-MS) spectra were recorded on an Agilent 1260 UPLC DAD 6530 Q-TOF mass spectrometer (Santa Clara, CA, USA). Nuclear magnetic resonance (NMR) data were acquired in pyridine-*d*_5_ on a Bruker DRX500 NMR spectrometer (Beijing, China) with ^1^H- and ^13^C-NMR observed at 500 and 125 MHz. Silica gel (200–300 mesh; Qingdao Marine Chemical Factory, China), RP-C18 (40–60 μm, Merck, Darmstadt, Germany) and Sephadex LH-20 (GE Healthcare Life Sciences, Shanghai, China) were used for column chromatography (CC). Thin-layer Chromatography (TLC) analysis was performed using on Silica gel GF254 plates (10–40 μm; Qingdao Marine Chemical Factory, Qingdao, China).

### 3.2. Plant Material

Herbs of *S. bigelovii* Torr. were collected from the beach of Yancheng, Jiangsu Province, China, in June 2013 and identified by Changqi Yuan, a professor at Institute of Botany, Jiangsu Province and Chinese Academy of Sciences, China. A voucher specimen (No. 13–03) was deposited with the Herbarium, Institute of Botany, Jiangsu Province and Chinese Academy of Sciences.

### 3.3. Extraction and Isolation

The air-dried powder of the herbs (5 kg) of *S. bigelovii* was extracted by 80% ethanol (3 × 150 L) at room temperature (25 °C) for 72 h each time. The solvent was evaporated under reduced pressure, leaving an extract (651 g) that was suspended in hot H_2_O (60 °C, 2000 mL). The suspension was partitioned successively with equal volumes of petroleum ether (PE), ethyl acetate (EtOAc) and *n*-BuOH. The *n*-BuOH-soluble fraction was concentrated under a vacuum to produce the *n*-BuOH extraction product (260.2 g, 0.52%). The *n*-BuOH-soluble fraction was subjected to silica-gel CC (1200 g, 200–300 mesh), eluted with a gradient of CH_2_Cl_2_–MeOH–H_2_O (95:5:0 to 0:100:0, *v*/*v*/*v*) to obtain eight fractions (Fr.1–Fr.8). Fr.2 (21.16 g) was separated through a Sephadex LH-20 CC [250 g, CH_2_Cl_2_:MeOH (1:1), *v*/*v*] to give five subfractions (Fr.2-1-Fr.2-5), and Fr.2-3 (1.5 g) was further subjected to RP-C18 CC (250 g, 50 µm) with MeOH:H_2_O (90:10–0:100 *v*/*v*) and RP-HPLC [MeOH:H_2_O (34:66, *v*/*v*)] to provide compound **1** (13.67 mg) and **2** (11.49 mg). Fr.3 (17.29 g) was separated by silica-gel CC (150 g, 200–300 mesh) with a gradient elution of EtOAc–MeOH (100:0 to 0:100, *v*/*v*) to obtain seven subfractions (Fr.3-1-Fr.3-8). Fr.3-1 and Fr.3-2 was purified using a Sephadex LH-20 CC with a CHCl_3_:MeOH eluent (1:1, *v*/*v*) to give compound **3** (12.43 mg) and compound **4** (17.39 mg), respectively. Fr.4 (37.53 g) was separated by RP-C18 (150 g, 50 µm) and eluted with MeOH–H_2_O (80:20→0:100, *v*/*v*) to produce nine subfractions (Fr.4-1-Fr.4-9). Fr.4-3 (153.93 mg) was purified by RP-HPLC [MeOH:H_2_O (70:30, *v*/*v*)] to give compound **5** (17.34 mg) and **6** (14.43 mg). Fr.4-4 (1037.61 mg) was purified by RP-HPLC [MeOH:H_2_O (45:55, *v*/*v*)] to give compound **7** (619.37 mg). Fr.4-5 (1025.47 mg) was purified by RP-HPLC using [MeOH:H_2_O (65:35, *v*/*v*)] to give compound **8** (521.92 mg) and **9** (16.33 mg). Fr.5 (17.61 g) was separated through Sephadex LH-20 CC [250 g, CH_2_Cl_2_:MeOH (1:1), *v*/*v*] to give three subfractions (Fr.5-1→Fr.5-3). Fr.5-2 (1.7 g) was further separated via RP-C18 CC (250 g, 50 µm) with MeOH–H_2_O (100:0→30:70, *v*/*v*) and purified by RP-HPLC [MeOH:H_2_O (74:26, *v*/*v*)] to provide compound **10** (16.57 mg) and **11** (14.47 mg).

Compound **11**: white amorphous powder, m.p. 205–206 °C, ${\left[\alpha \right]}_{\text{D}}^{20}$ +27.9 (*c* 0.479, MeOH), IR *v*_max_ cm^−1^: 3412, 1705 and 1645, HREI-MS (negative ion mode) *m/z*: 629.3261 [M − H]^−^ (calc. for C_35_H_49_O_10_, 629.3285). ^1^H-NMR, ^13^C-NMR, DEPT, ^1^H-^1^H COSY and HMBC data see Table 1.

### 3.4. Fungus Strains and Positive Controls

The pathogens, Alternaria alternata, A. solani, Botrytis cinerea, Colletotrichum gloeosporioides, Fusarium graminearum, F. verticilloides, Thanatephorus cucumeris, Sclerotinia sclerotiorum, used in the bioassays were identified by Pingping Song, at Institute of Botany, Jiangsu Province and Chinese Academy of Sciences, China. Positive controls Amphotericin B (AMB) was purchased from Sigma-Aldrich (St Louis, MO, USA).

### 3.5. Antifungal Assays in Vitro

Inhibitory activity against the hyphal growth of pathogenic fungus was determined using a growth rate method [12]. A test solution with the required concentration can be made by dissolving the sample in DMSO. 1 mL of this solution and 24 mL of PDA molten agar medium were mixed thoroughly and distributed equally into 3 petri dishes (6 cm diameter). DMSO, without any sample, was processed similarly to produce the control culture media. Then a toxic culture media can be made. The discs of hyphae, which was cut from the edge of the colony and had been incubated in advance, was put downward in the middle of culture medium’s surface. By the least-squares method, we can acquire the discs, regression equations, EC_50_ and 95% confidence intervals.

## 4. Conclusions

In the present study, a new nortriterpenoid saponin (**11**) was isolated from the herbs of *S. bigelovii*, and it has a significant inhibitory effect on plant pathogenic fungus. Comparison of the structures of compound **3**, **4**, **5**, **6**, **7**, **10** and **11** with those of inactive compounds, suggested that the aldehyde group at C-23, the carboxyl group at C-28 and the ester group at C-6’ of glucuronopyranosyl moiety might have contributed to the inhibition of the growth of *C. gloeosporioides* synergistically.

## Supplementary Materials

Supplementary materials can be accessed at: http://www.mdpi.com/1420-3049/20/11/19695/s1.
