# Supplementary material for: Triterpenoids from the Herbs of Salicornia bigelovii
Source: Molecules. 2015 Nov 12;20(11):20334–40. doi: 10.3390/molecules201119695 (PMC6332287; doi:10.3390/molecules201119695)
Supplement: Supplementary file 1 [file molecules-20-19695-s001.pdf]

Supplementary Materials: Triterpenoids from the Herbs of *Salicornia bigelovii*

Yu Shan, Huan Li, Fuqin Guan, Yu Chen, Min Yin, Ming Wang, Xu Feng and Qizhi Wang

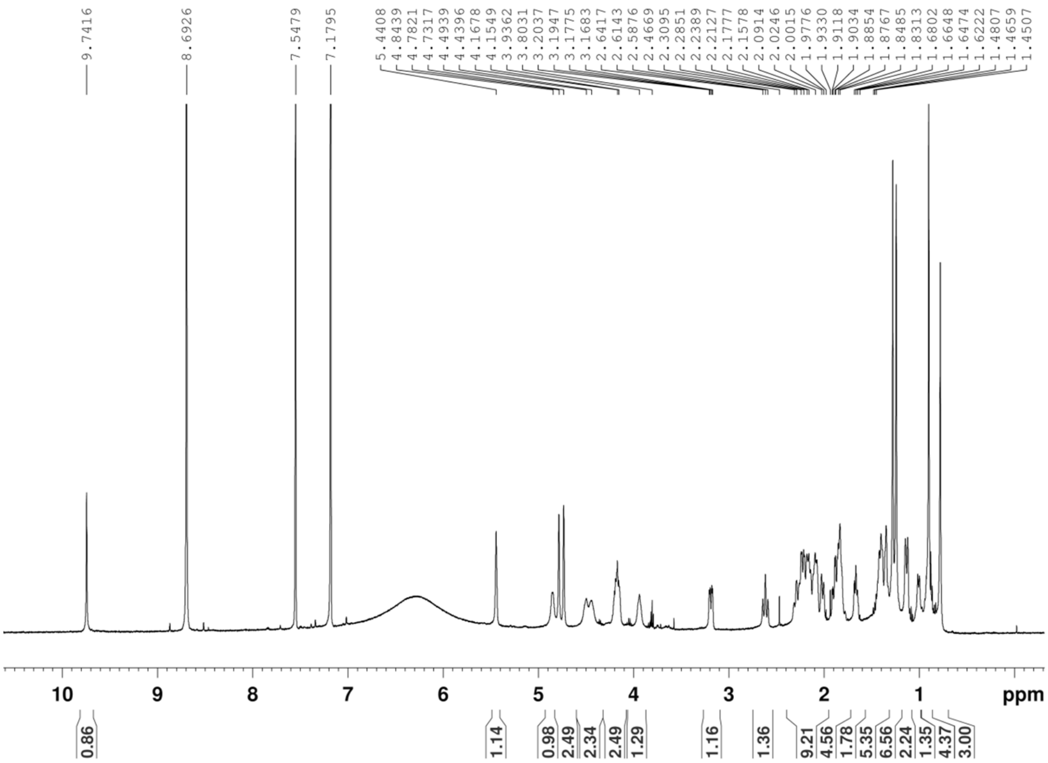

Figure S1. ^{1}H-NMR spectrum of Salibige A (**11**) (pyridine-d_5, 500 MHz, δ ppm).

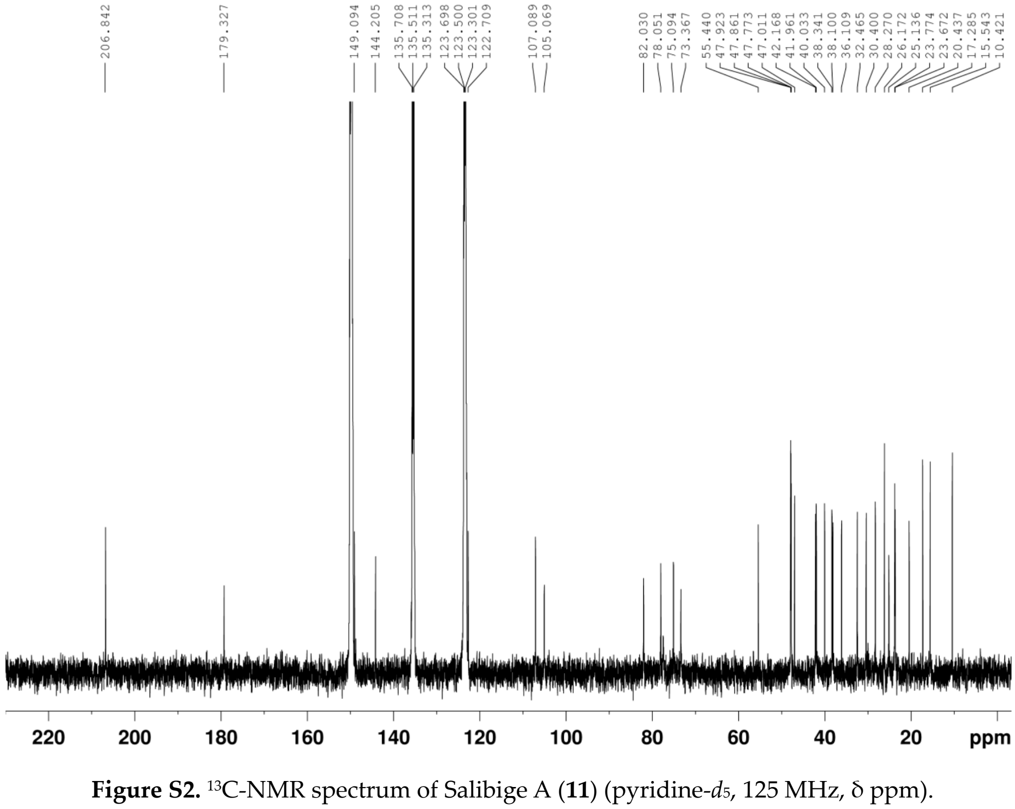

Figure S2. ^{13}C-NMR spectrum of Salibige A (**11**) (pyridine-d_5, 125 MHz, δ ppm).

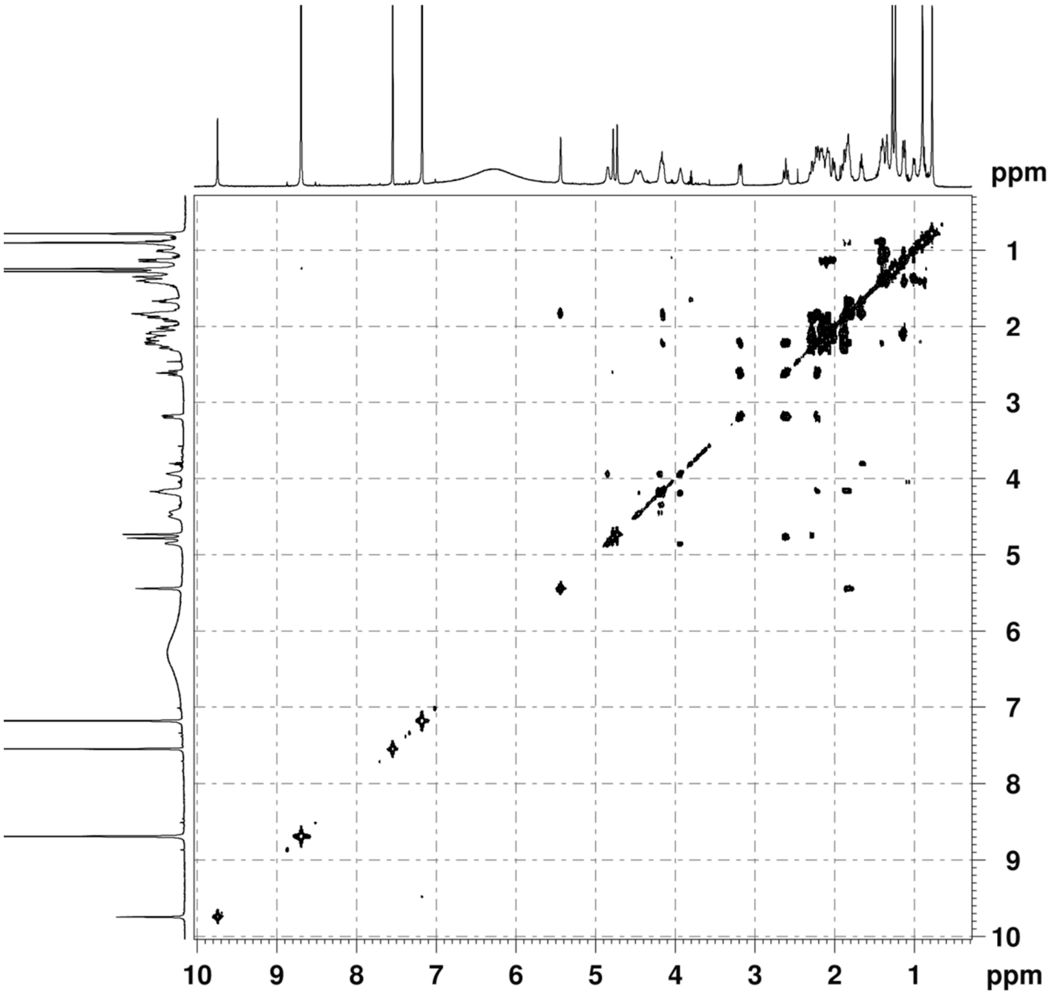

Figure S3. ^{1}H-^{1}H COSY spectrum of Salibige A (**11**) (pyridine-d_5, δ ppm).

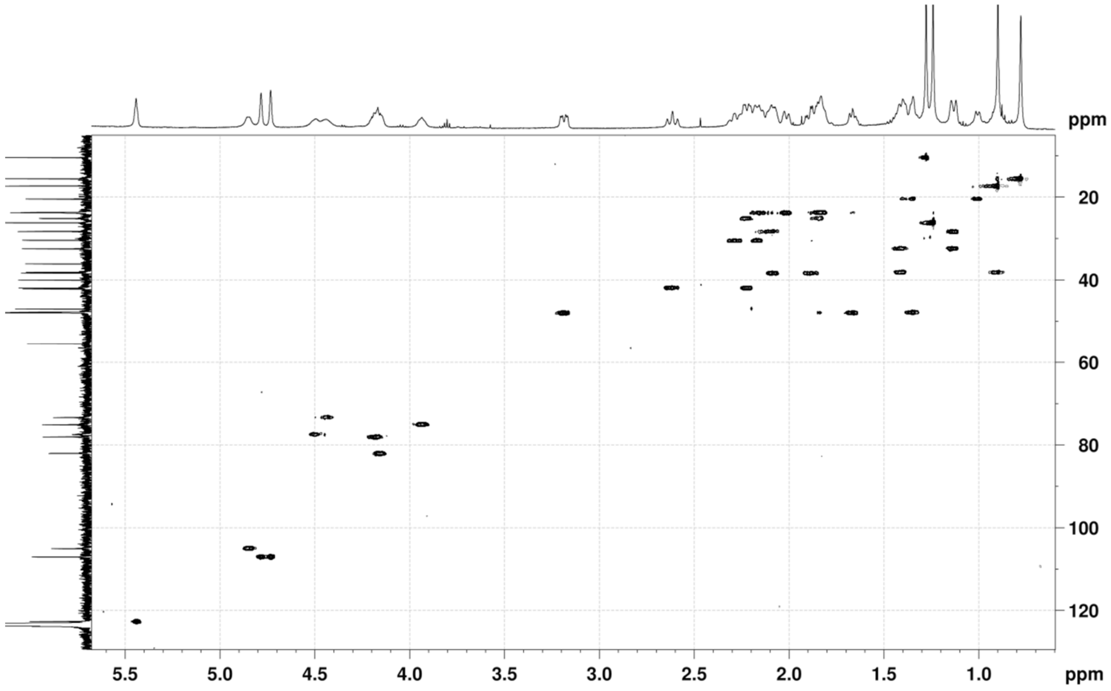

Figure S4. HMQC spectrum of Salibige A (**11**) (pyridine-d_5, δ ppm).

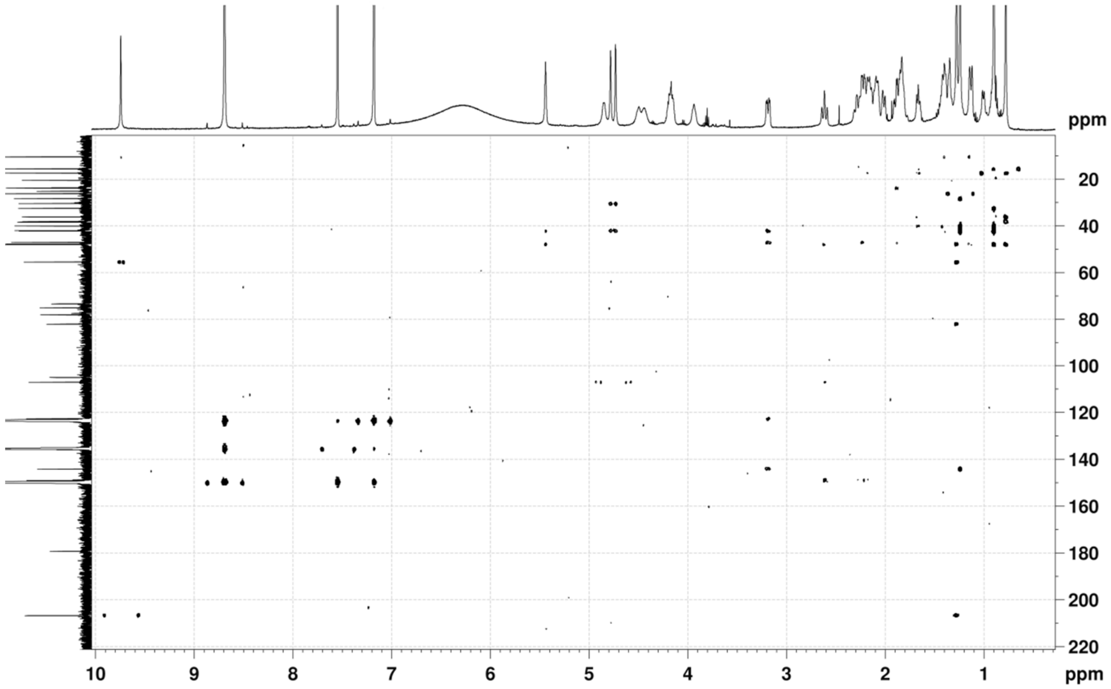

Figure S5. HMBC spectrum of Salibige A (**11**) (pyridine-d_5, δ ppm).

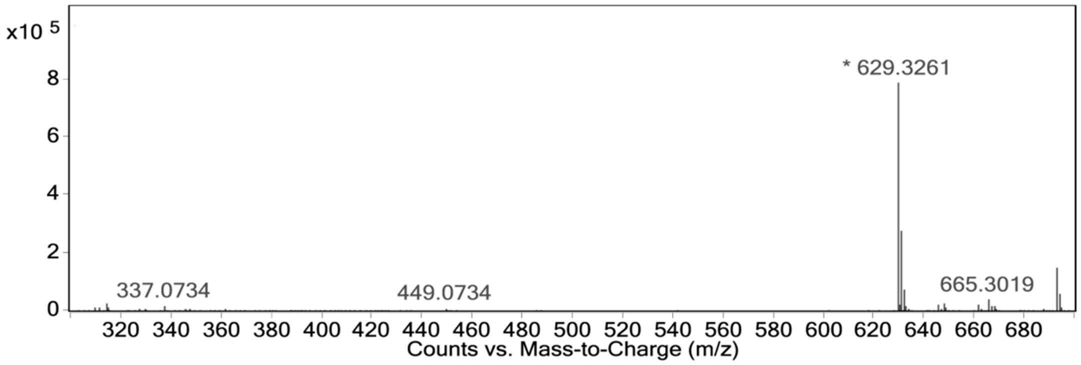

Figure S6. Q-TOF HRMS spectrum of Salibige A (**11**).

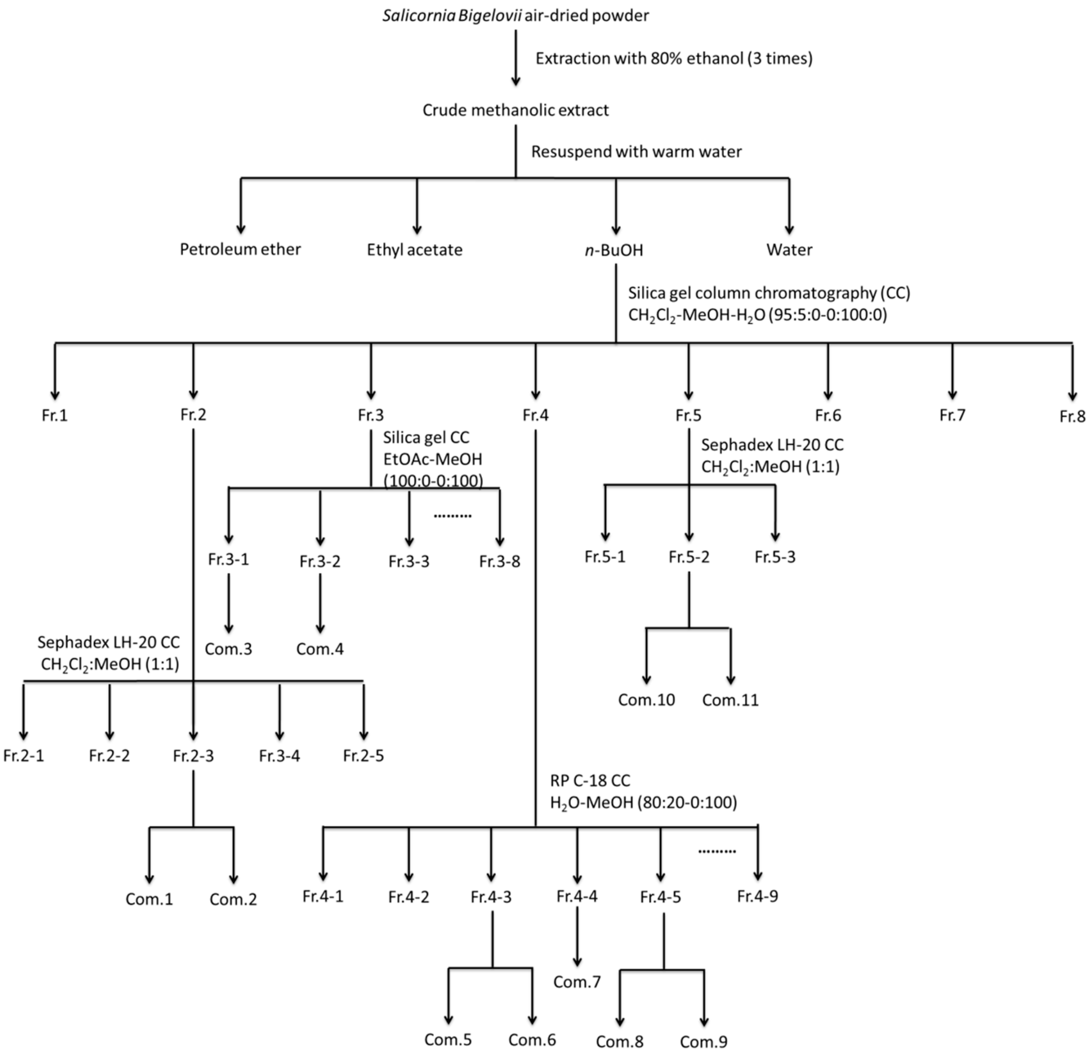

Figure S7. Extraction scheme for the isolation of antifungal saponins from *S. Bigelovii.*
